# Loss of Cep72 affects the morphology of spermatozoa in mice

**DOI:** 10.3389/fphys.2022.948965

**Published:** 2022-10-07

**Authors:** Zhen Chen, Yating Xu, Dupeng Ma, Changrong Li, Ziqi Yu, Cong Liu, Tingyu Jin, Ziye Du, Zejia Li, Qi Sun, Yumin Xu, Rong Liu, Yuerong Wu, Mengcheng Luo

**Affiliations:** ^1^ Hubei Provincial Key Laboratory of Developmentally Originated Disease, TaiKang Medical School (School of Basic Medical Sciences), Wuhan University, Wuhan, China; ^2^ Center for Animal Experiment, Wuhan University, Wuhan, China

**Keywords:** Cep72, centrosome, spermiogenesis, fertility, sperm flagellum

## Abstract

The centrosome regulates mammalian meiosis by affecting recombination, synapsis, chromosome segregation, and spermiogenesis. Cep72 is one of the critical components of the centrosome. However, the physiological role of Cep72 in spermatogenesis and fertility remains unclear. In this study, we identify Cep72 as a testis-specific expression protein. Although *Cep72* knockout mice were viable and fertile, their sperms were morphologically abnormal with incomplete flagellum structures. Transcriptome analysis reveals significant differences in six genes (*Gm49527*, *Hbb-bt*, *Hba-a2*, *Rps27a-ps2*, *Gm29647*, and *Gm8430*), which were not previously associated with spermatogenesis. Overall, these results indicate that Cep72 participates in regulating sperm morphology and yet is dispensable for fertility in mice.

## Introduction

The centrosome is the main microtubule organization center (MTOC) composed of a centriole pair and the pericentriolar material (PCM). The centrosome is crucial for maintaining genomic integrity by participating in the regulation of organelle positioning, cell motility, intracellular transport, and mitotic spindle assembly (Bornens, 2012; Lawo et al., 2012; Sonnen et al., 2012). Disruptions in the number and structure of centrosomes may be one of the fundamental causes of cancer and disease (Nigg and Raff, 2009; Chan, 2011; Jaiswal and Singh, 2020; Remo et al., 2020). The centrosome also plays a critical role in meiosis, which mainly involves centrosome remodeling in sperm and centriole disappearance in the oocyte (Avidor-Reiss and Fishman, 2019; Gonczy and Hatzopoulos, 2019). Disruption of the centrosome in germ cells may lead to infertility in mammals.

Recently, it has been found that the abnormality of Cep family proteins such as Cep135, Cep131, Cep63, Cep164, and Cep70 can lead to abnormal gametogenesis and infertility in animals and humans (Hall et al., 2013; Kumar et al., 2013; Marjanovic et al., 2015; Sha et al., 2017; Avidor-Reiss et al., 2020; Hoque et al., 2021; Liu et al., 2021). Cep72, one of the Cep family proteins, is also a PCM protein (Kumar et al., 2013). Overexpression or knockdown of *Cep72* in somatic cells impaired the process of mitosis, such as abnormal spindle formation, disturbed microtubule organization, and generated wrong chromosomal alignment (Oshimori et al., 2009; Li et al., 2019b; Ni et al., 2020). Current studies of Cep72 mostly focus on somatic cells. However, the function of *Cep72* in mammalian gametogenesis is still unclear.

Here, we found that Cep72 is specifically expressed in mouse testis. Then, we generated a *Cep72* knockout (KO) mouse line *via* the CRISPR/Cas9 system. Interestingly, *Cep72*-null male mice had no detectable abnormalities in the meiotic process, and both male and female *Cep72* KO mice were fertile. However, loss of *Cep72* led to abnormal morphology of sperm heads and flagella since typical centrosomes remodel into atypical centrosomes and flagella during spermiogenesis (Avidor-Reiss and Fishman, 2019) and aberrations in centrosome remodeling lead to defective sperm morphology. Our findings indicate that Cep72 may be critically involved in centrosome remodeling. Overall, we demonstrate that the absence of *Cep72* partially affects the morphology of sperms but does not impair meiosis and fertility in mice.

## Materials and methods

### Generation of *Cep*72 KO mice and genotyping

We generated a *Cep72* KO mouse line with cytoplasmic injection using CRISPR/Cas9 gene-edited technology (Yang et al., 2014). Two small guide RNAs (sgRNAs) were designed in exon3 of *Cep72*, with the sequences of sgRNA1:5′-TCTTCACAACGGGGTTAAGCCGG-3′ and 5′-sgRNA2: GGA​GCA​TGT​GCA​CGA​CAA​ACA​GG-3′. The mouse genotypes were confirmed by PCR with primers *Cep72*-JD-F (5′-TAC​AGT​ATC​TGG​TCT​CCT​TGG-3′) and *Cep72*-JD-R (5′-CAT​GGA​ACA​GCA​GCA​TAA​TC-3′), and the products were WT 265bp and KO 218bp. All animal experiments in the study complied with regulatory standards approved by the Institutional Animal Care and Use Committee of Wuhan University.

### Antibody production

The Cep72 (333-613aa) cDNA fragment of the mouse was cloned in the pET-42 b vector and expressed in *E. coli* strain DE3. The GST-Cep72 (333-613aa)-8xHis antigen (66.5 kD) was purified with an Ni-NTA resin and used to immunize the rabbit. Rabbit polyclonal antibody serum was purified by the GST-Cep72 (333-613aa)-8xHis antigen and stored in 100 mM glycine (PH 8.0) at -80°C (Luo et al., 2013).

### Fertility assessment

The fertility of *Cep72* KO male mice was tested by being mated with WT C57BL/6N female mice. The fertility of *Cep72* KO female mice was tested by being mated with WT C57BL/6N male mice. In short, one 2–3-month-old KO male mouse was mated with one 8–10-week-old WT female mouse, and one 8–10-week-old KO female mouse was mated with one 10–12-week-old WT male mouse for at least three months. The litter sizes, sex ratio, and birth intervals by every group of males or females were recorded.

### Sperm count and staining

To count the sperm number, 10-week-old male mice were sacrificed by cervical dislocation. One side of cauda epididymis from each mouse was removed and cut into small pieces in a 1.5-ml tube, adding 1 ml 1x PBS. The tubes were incubated at 37°C for 30 min in an incubator to allow the sperm to swim out into PBS (Gao et al., 2020). Then, sperm counts were conducted by using the hemocytometer (Jiang et al., 2017).

To evaluate and observe the sperm morphology, we stained the sperm using PNA (RL-1072, Vector Laboratories, United States) and DAPI (ZLI-9557, ZSGB-BIO, China) combined with bright-field observation. This experiment was performed using an M2 microscope (Zeiss, Germany).

## RT-PCR

TRIzol reagents were used to extract the total RNA from different tissues of mice, and reverse transcription was performed using a PrimeScript RT reagent kit (TaKaRa, RR047A). For RT-PCR, the primers of *Cep72* were designed, and *ACTB* was used as a reference gene (*Cep72*-F: 5′- TAC​AGT​ATC​TGG​TCT​CCT​TGG-3′, *Cep72*-R: 5′- TCA​GTG​AAT​CTT​CTG​GGG​CAA​AAT​G-3′, product 258bp; *ACTB*-F: 5′- AGG​CTG​TGC​TGT​CCC​TGT​AT-3′, *ACTB*-R: 5′-CTC​TCA​GCT​GTG​GTG​GTG​AA-3′, product 208bp).

### Western blot analysis

Total proteins were extracted from different tissues with RIPA buffer adding protease inhibitor cocktail (PIC) (Solarbio, China). Western blot was performed as reported previously (Li et al., 2019a). Cytoplasmic and nuclear protein fractions of the testis were isolated. Briefly, 200 mg adult testes were homogenized in 1 ml buffer 1 (250 mM sucrose, 10 mM Tris–HCl, 10 mM MgCl2, and 1 mM EDTA, pH 8.0) plus 1x PIC with 50 strokes of a tight-fitting pestle on ice, and then centrifuged at 12000 rpm for 1 min at 4°C. The supernatant mainly contained cytoplasmic proteins. Then, the pellet was suspended with 1 ml buffer 1 plus 0.25% NP-40, 0.1% Triton X-100, and 1 x PIC. The suspension was homogenized again with 40 strokes and centrifuged at 100xg for 30s at 4°C. The supernatant mainly contained nuclear proteins. The primary antibodies for Western blotting included anti-Cep72 (1:500; generated by our laboratory), anti-ACTB (1:2000, GB12001, Servicebio, China), anti-GAPDH (1:20000, AC033, ABclonal, China), anti-H3 (1:3000, 17168-1-AP, Proteintech, United States), and anti-Scml2 (1:500; donated by P. Jeremy Wang’s laboratory).

### Histology

Testes or ovaries were fixed overnight in 4% paraformaldehyde (PFA) or Bouin’s fixative (Sigma-Aldrich, United States) at 4°C. Fixed tissues were subjected to embedding in paraffin and sectioning. Testis or ovary sections were stained with hematoxylin and eosin (H&E) (Luo et al., 2013).

### Spermatocyte spreading and immunostaining

Spermatocyte chromosome was prepared as previously reported (Peters et al., 1997). Spread nuclei were stained with primary antibodies against synaptonemal complex protein 3 (SYCP3, a marker of SC) (1:200, prepared by our laboratory) and γH2AX (a marker of DNA damage and repair) (1:500, 05-636, Millipore, United States).

### Metaphase spermatocyte spreading and staining

Metaphase spermatocyte spreading was performed as previously described (Jiang et al., 2017). The testis was removed from the tunica albuginea and cut into pieces. Then, 2.2% (w/v) trisodium citrate dehydrate was added. Single-cell suspensions were treated with 0.9% (w/v) trisodium citrate dihydrate and fixed with methanol and acetic acid (3:1; v/v). After being washed, metaphase spermatocyte chromosomes were stained with Giemsa and observed using a microscope (ZEISS M2, Germany).

### Transmission electron microscope

Briefly, the sperm from cauda epididymis were obtained in DMEM, washed three times with 1x PBS (pH 7.4), and then fixed in 2.5% glutaraldehyde solution. After washing using 0.1 M PB, the sperm were fixed with 1% O_S_O_4_, dehydrated, and then embedded in a resin. Ultrathin sections were stained with 2% uranyl acetate and lead citrate and observed using TEM (HT7700, Hitachi, Japan) (Yuan et al., 2015).

### RNA-seq

Testes from 4-month-old WT and *Cep72* KO mice were collected and then sent to Novogene for sequencing. Briefly, total RNA was extracted, and the RNA integrity and total volume were assessed by using the Agilent 2100 bioanalyzer. Then, the mRNA with polyA tails was enriched using oligo (dT) magnetic beads, and the resulting mRNA was subsequently randomly cut into short segments in fragmentation buffer. The fragmented mRNA was used as a template, and random oligonucleotides were used as primers to synthesize the first strand of cDNA in the M-MuLV reverse transcriptase system, followed by degradation of the RNA strand with RNaseH and synthesis of the second strand of cDNA with dNTPs under the DNA polymerase I system. The purified double-stranded cDNAs were repaired at the end, and A-tail was added and connected to the sequencing adapter. Next, the cDNAs of 370–420bp were screened by AMPure XP beads and amplified by PCR. Finally, the PCR products were purified again using AMPure XP beads to obtain the RNA library. The Qubit 2.0 Fluorometer and Agilent 2100 bioanalyzer were used to detect the quality of the RNA library. The qualified libraries were sequenced in Illumina to generate 150 bp paired-end reads. The clean data were obtained, and HISAT2 v2.0.5 was used to construct an index of the reference genome and compared paired-end clean reads to the reference genome. Differential expression was analyzed between WT and KO groups using DESeq2 software. After correction, padj values and log2 fold-change were used as thresholds for significant differential expression. The data have been deposited at SRA, and the BioProject ID/Accession No. is PRJNA835886.

### Statistical analysis

GraphPad Prism 8.0.2 (GraphPad Software Inc., San Diego, CA, United States) and R (https://www.r-project.org/) were used to calculate all data and draw graphs. The data were presented as mean ± SEM, and statistical analysis was performed using Student’s *t*-test. A *p*-value < 0.05 was considered significant.

## Results

### Cep72 protein is specifically expressed in mouse testis

**FIGURE 1:**
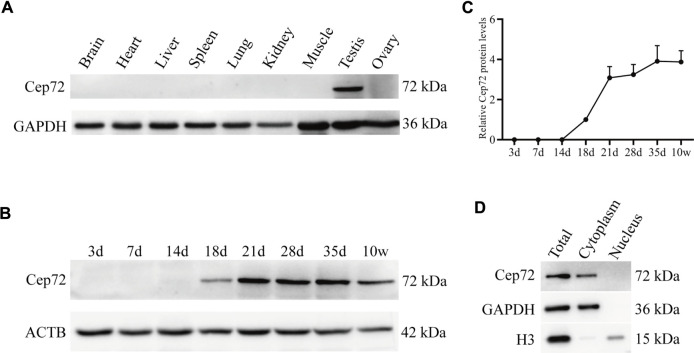
Expression patterns of Cep72 protein in mice. **(A)** Western blot displayed the protein profiling of Cep72 in multiple tissues. GAPDH was used as an internal control. **(B)** Western blot showed the protein expression of Cep72 in testes of mice with various postnatal days. *ACTB* served as an internal control. **(C)** Relative Cep72 protein levels were quantified for **(B)**. Data were provided as mean ± SEM, n = 3. **(D)** Location of the Cep72 expression was detected by WB. Total was the whole cell lysate. GAPHD served as a cytoplasmic marker, and H3 served as a nuclear marker.

To determine the function of Cep72 in fertility, we first analyzed its expression profile in multiple tissues from adult mice by RT-PCR and Western blot, respectively. The results showed that *Cep72* mRNA is highly expressed in the testis (Supplementary Figure S1A). To detect the protein level of Cep72, we generated a rabbit polyclonal antibody with the purified Cep72 antigen (333-613aa) (Supplementary Figure S1B). Interestingly, Cep72 protein is specifically expressed in the testis only (Figure 1A). To further explore the expression pattern of Cep72 during spermatogenesis, we measured the Cep72 protein from postnatal day 3 (3 d) to 10 weeks (10w) and observed that Cep72 was initially expressed at 18 d and increased to the highest expression at 35 days (Figures 1B and C). Furthermore, Cep72 is a cytoplasmic protein (Figure 1D). Analysis of single-cell RNA-seq (scRNA-seq) data (GSE107644) (Chen et al., 2018) found that *Cep72* is predominantly expressed in pro-meiosis spermatocytes (Lep-MII) and post-meiosis spermatid (RS2-RS6) of mouse testis (Supplementary Figure S1C). Meanwhile, the scRNA-seq data (GSE106487) (Wang et al., 2018) of human testis shows that *Cep72* is mainly expressed in spermatocytes (Lep-Zgy) (Supplementary Figure S1D). These data show that *Cep72* gene is consistently expressed in male germ cells. In summary, these data suggest that Cep72 plays an important role in spermatogenesis and fertility.

### Normal fertility in *Cep72*-deficient mice

**FIGURE 2:**
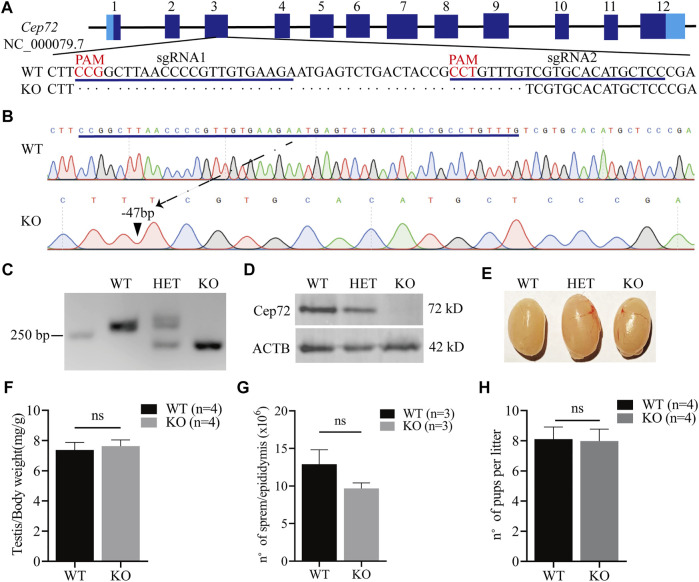
Loss of Cep72 does not impair the fertility of mice. **(A)** Targeting strategy of *Cep72* KO mice using CRISPR/Cas9 technology. Boxes are exons of the *Cep72* gene (NC_000079.7), while the dark and light blue boundaries represent where the start (1) and stop (12) codons are located. The blue lines display two designed sgRNAs, and the red mark shows the PAM (protospacer adjacent motif) of sgRNAs. **(B)** Results of Sanger sequencing showing 47 bp deletion from *Cep72* KO and WT mice. The blue line represents the missing sequence. The dashed line and black triangle indicate the position of the lost sequence in *Cep72* KO mice. **(C)** Representation of PCR genotyping, and 250 bp is the location of the marker. WT, wild type (265 bp); HET, heterozygous; KO, knockout (218 bp); 2% agarose gel. **(D)** Western blot results demonstrating the Cep72 protein expression in the testis of 10-week-old WT, HET, and *Cep72* KO mice. *ACTB* served as an internal control. **(E)** Morphology of the testis of 10-week-old WT, HET, and *Cep72* KO mice. **(F)** Testis to body weight ratio of 10-week-old WT and *Cep72* KO mice. Data are presented as mean ± SEM, n = 4; ns: no significant difference. **(G)** Sperm number per epididymis of 10-week-old WT and *Cep72* KO mice. Data are presented as mean ± SEM, n = 3; ns: no significant difference. **(H)** Number of pups per litter from WT and *Cep72* KO male mice. Data are presented as mean ± SEM, n = 4; ns: no significant difference.

To reveal the physiological function of *Cep72* in fertility, we generated the *Cep72* knockout (KO) mice by using the CRISPR/Cas9 system. Two sgRNAs were designed for targeting the exon3 (Figure 2A). We obtained a strain of mice with 47 bp deleted in exon3 of the *Cep72* gene by Sanger sequencing analysis (Figure 2B). The deletion resulted in an open reading frameshift and generated a premature termination codon. Our PCR results confirmed a 47 bp deletion in the *Cep72* genome sequence (Figure 2C). Furthermore, the Cep72 protein was completely undetectable in the testis of *Cep72* KO adult mice (Figure 2D). The *Cep72* KO mice were viable and had no observable physiological defects. We did not find any significant abnormalities in morphology and weight in the testis of *Cep72* KO mice compared with wild-type (WT) mice (Figures 2E and F). We observed a mild but insignificant decrease in sperm number in *Cep72* KO mice compared with WT mice (Figure 2G). The fertility test confirmed that the males and females of *Cep72* KO mice did not show substantial change in the number of pups per litter compared to those of the WT control group (Figure 2H and Supplementary Figure S2A). Together, these results indicate that *Cep72* is not essential for fertility in male and female mice.

### *Cep72* is dispensable for the meiosis process during spermatogenesis

**FIGURE 3:**
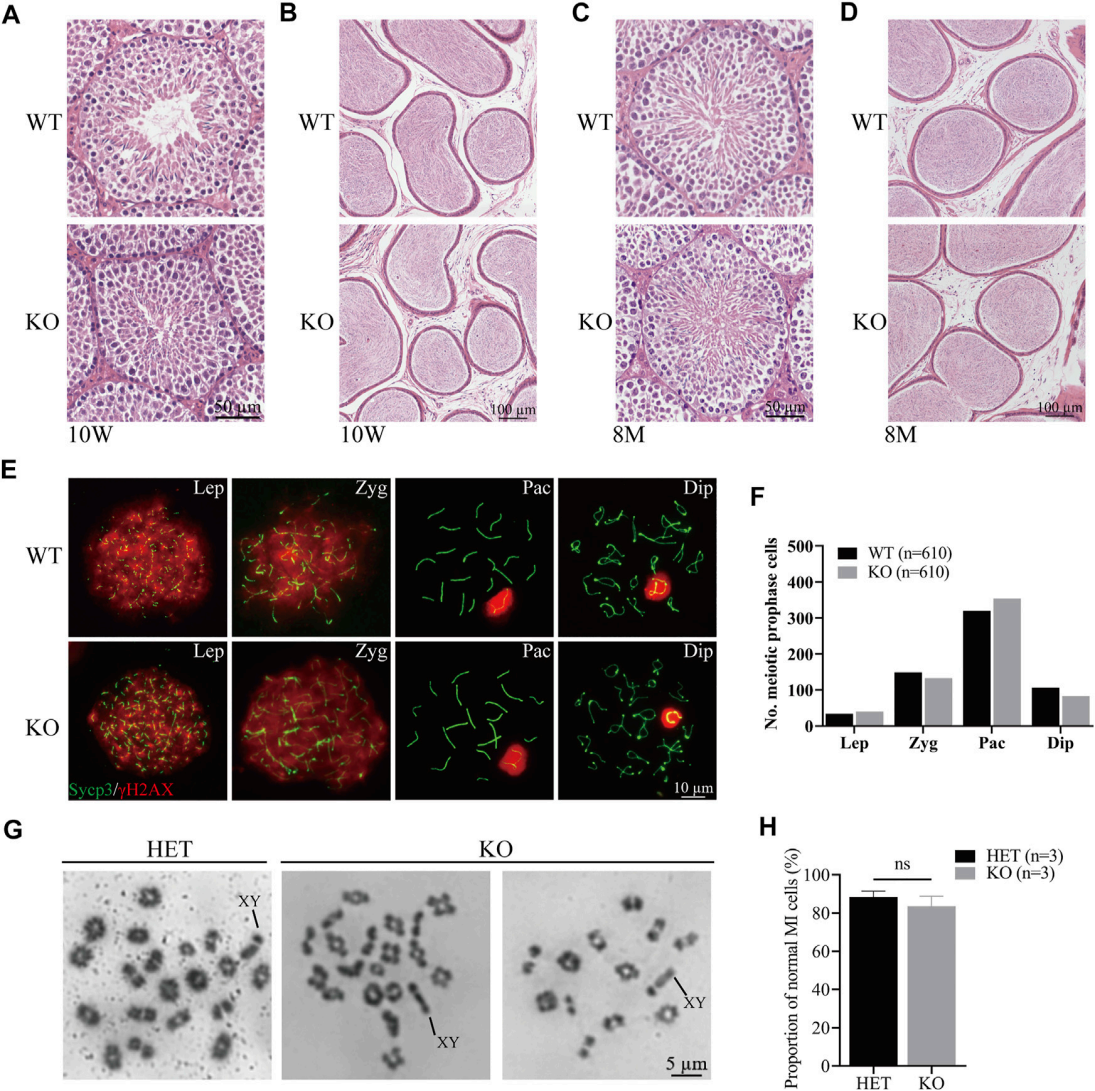
Meiosis process during spermatogenesis is normal in *Cep72* KO mice. **(A** and **B)** H&E staining of the testis and epididymis from 10-week-old WT and *Cep72* KO mice, respectively, bar = 50 and 100 μm. **(C** and **D)** H&E staining of the testis and epididymis from 8-month-old WT and *Cep72* KO mice, respectively, bar = 50 and 100 μm. **(E)** Representative spread spermatocytes stained for Sycp3 (green) and γH2AX (red) from WT and *Cep72* KO mice, bar = 10 μm. Lep, leptotene; Zyg. zygotene; Pac, pachytene; Dip, diplotene. **(F)** Number of meiotic prophase cells at Lep, Zyg, Pac, and Dip stages from WT and *Cep72* KO mice (three mice per genotype used, 610 WT and 610 *Cep72* KO cells scored). **(G)** Analysis of chiasmata in metaphase I (MI) spermatocytes from HET and *Cep72* KO mice at 4 weeks. The black lines indicate the XY chromosome. Scale bar = 5 μm. **(H)** Quantification of chiasmata MI cells. The data are from three mice per genotype; ns: no significant difference.

To evaluate the role of *Cep72* in the meiosis process, we performed the histological analysis and found that the structural integrity of seminiferous tubules and sperm concentration in the cauda epididymis were normal in 10-week-old and 8-month-old *Cep72* KO mice (Figures 3A–D). Similarly, the H&E staining of the ovaries of 6-month-old *Cep72* KO mice showed no significant defects in follicular development either (Supplementary Figure S2B). Furthermore, spermatocyte chromosome spreading showed normal synapsis, and the DSBs were processed with no observable defects in *Cep72* KO mice (Figure 3E). Compared with WT mice, there was no significant retardation or deceleration in prophase I spermatocytes (Lep-Dip) (Figure 3F). Next, we assessed the normal metaphase I spermatocytes and found no significant change in the *Cep72* KO mice compared with the heterozygote (HET) mice (83.60% vs. 88.42%, *p* > 0.05) (Figures 3G and H). Overall, our results indicate that the absence of *Cep72* has minimal influence on the meiosis process during spermatogenesis.

### *Cep72* maintains sperm morphological integrity

**FIGURE 4:**
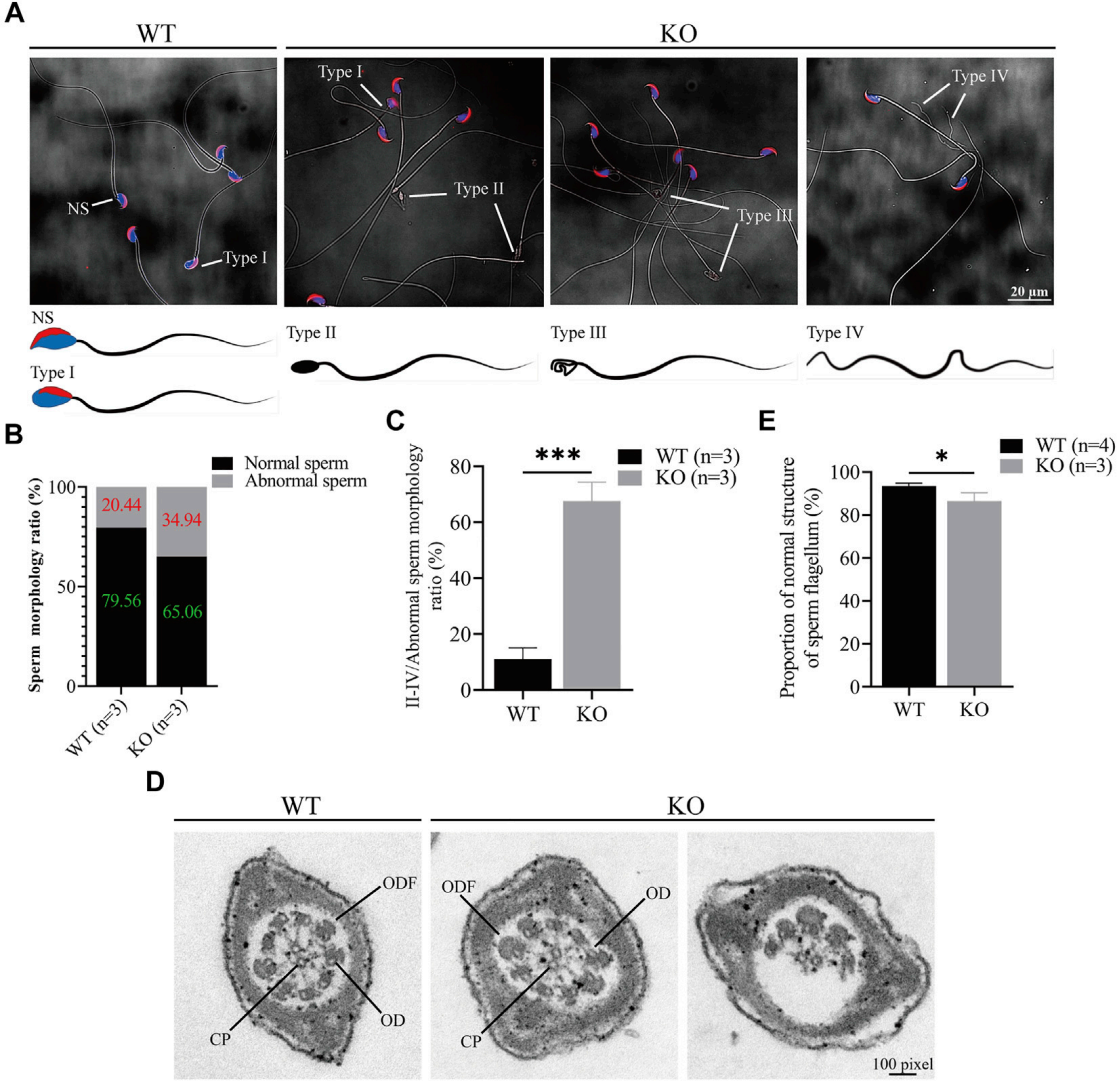
Loss of *Cep72* impairs the sperm morphology of mice. **(A)** Representative sperm morphology and images of sperm of WT and *Cep72* KO mice. PNA (red) and DAPI (blue) indicated the acrosome and nucleus of sperm, respectively. NS: normal sperm, Type I (abnormal head had PNA and DAPI), Type II (rod head had no PNA and DAPI), Type III (hollow head had no PNA and DAPI), and Type IV (no head and curved flagellum) were abnormally shaped spermatozoa. Bar = 20 μm. **(B)** Stacking diagram showing the ratio of abnormal sperm from WT and *Cep72* KO mice, n = 3. **(C)** Percentages of types II–IV abnormal sperm to all abnormal sperm. Data are presented as mean ± SEM, n = 3, ***p < 0.001. **(D)** Sperm flagellum detection by TEM, bar = 100 pixel. ODF, outer dense fiber; OD, outer doublet microtubule of the axoneme; CP, central pair microtubules. **(E)** Quantification of normal sperm flagellum for **(D)**. The data are from four WT and three *Cep72* KO mice, *p < 0.05.

Since the sperm count decreased in the *Cep72* KO mice, we next investigated sperm morphology. Interestingly, we observed a marked increase in the number of morphologically abnormal spermatozoa in *Cep72* KO mice (34.94% vs. 20.44%, *p* < 0.001) (Figures 4A and B). We classified the abnormal sperms into four types according to their morphology and showed them as images (Figure 4A): NS was normal sperm; type I showed incorrect head shape with PNA (acrosome marker) and DAPI (nucleus marker) staining; type II displayed rod head with no acrosome and nucleus; type III showed hollow head with no acrosome and nucleus; and type IV had no head and curved flagellum. Further analysis found that the abnormal sperm morphology of types II and IV was significantly increased in *Cep72* KO mice compared to WT mice (67.57% vs. 11%, *p* < 0.001) (Figure 4C). Although no head sperm were also present in *Cep72* KO mice, there was no significant difference compared to WT mice, while type IV sperm were barely observed in WT mice. Next, to explore the underlying mechanism of the abnormal morphology, we performed an ultrastructural test under the transmission electron microscope (TEM) and found a significant increase in the rate of structural abnormalities in the sperm flagellum in *Cep72* KO mice compared with WT mice (13.51% vs. 6.53%, *p* < 0.05) (Figures 4D,E). These abnormal flagella showed that ODF (outer dense fiber), CP (central pair microtubules), and OD (outer doublet microtubule) were partially absent in the principal piece of some sperms.

To examine whether the loss of *Cep72* affects the transcriptome and whether there are compensatory genes of *Cep72* family members, we performed RNA-seq analysis using testes from adult *Cep72* KO and WT mice. The results showed no overall changes in the global transcriptome. However, six transcripts (*Gm49527*, *Hbb-bt*, *Hba-a2*, *Rps27a-ps2*, *Gm29647*, and *Gm8430*) were significantly different in *Cep72* KO testes compared with WT testes (Supplementary Figures S2C,D). None of these genes have been reported to be essential for spermatogenesis. Together, these results suggest that the Cep72 participates in the regulation of sperm morphogenesis.

## Discussion

Mammalian spermatogenesis is a highly organized biological process that involves mitosis, meiosis, and spermiogenesis (De Kretser et al., 1998). The correct assembly of centrosomes is essential in spermatogenesis (Avidor-Reiss et al., 2020). The function of Cep72, a member of the pericentriolar material (PCM) family, in mammalian fertility and spermatogenesis is unknown. Through analyzing the scRNA-seq data (GSE106487) (Wang et al., 2018) of the human testis, we found that *Cep72* is mainly expressed in spermatocytes (Lep-Zgy) (Supplementary Figure S1D). We also found that *Cep72* is predominantly expressed in pro-meiosis spermatocytes (Lep-MII) and post-meiosis spermatids (RS2-RS6) in the mouse testis (Supplementary Figure S1C). Since the function of *Cep72* in the reproductive process has not been reported, we generated a *Cep72* KO mouse model and provided a reference for future studies of human *Cep72*.

Notably, we observed that Cep72 is a testis-specific protein in mice, though *Cep72* mRNA is enriched in the testis but also expressed in many other tissues (Figure 1A and Supplementary Figure S1A). A potential explanation (White et al., 2004) is the different correlation between mRNA and protein expression in various tissues due to the effects of differential regulations of mRNA translation, mRNA silencing, and protein stability. Therefore, even though scRNA-seq data suggest that *Cep72* mRNA expression patterns are not identical in mice and humans, the Cep72 protein expression pattern in mice might still be conserved in human. In mice, the Cep72 expression appeared around postnatal day 18 and gradually increased thereafter (Figures 1B,C). We detected almost no Cep72 expression in the testes of mice at 3, 7, and 14 days after birth, implying that Cep72 is not expressed in spermatogonia or somatic supporting cells, such as Sertoli cells and Leydig cells but is a mouse spermatogenic-specific protein. These data strongly suggest that *Cep72* plays an important role in mouse fertility and spermatogenesis. Surprisingly, both male and female *Cep72* KO mice were viable and fertile (Figure 2H and Supplementary Figure S2A). Given that Cep72 is a negative regulator of Brca1 (Luddecke et al., 2016), we examined the DSB repair but did not find obvious change in spermatocytes (Figure 3E). This result implies that the deficiency of Cep72 may have affected the upregulation of Brca1, which promoted the repair of DNA damage and therefore did not result in abnormal DSB damage repair in spermatocytes. Previous reports showed that defects in centrosomes (loss of *Cep63*) led to SC entanglements and delayed the early stages of meiotic prophase I to later stages (Marjanovic et al., 2015). However, we detected normal SC formation and meiotic prophase I procession in spermatocytes of *Cep72* KO mice (Figures 3E,F). Recently, it was reported that loss of *Cep70* had no effect on the prophase of meiosis I but caused the abnormal formation of flagella and acrosomes during spermiogenesis in mice (Liu et al., 2021). Consistently, in our study, we found that the deletion of *Cep72* disturbed the abnormal morphology in some spermatozoa (Figure 4A). In addition, some sperm flagellum structures were incomplete in *Cep72* KO mice (Figure 4D). These results suggest that loss of Cep72 does not affect fertility but may lead to some degree of defects in spermiogenesis in mice.

As meiosis proceeds during mammalian spermatogenesis, the centrosome reassembles and forms an atypical centriole (Avidor-Reiss and Fishman, 2019). A normal spermatozoon is composed of a head and a tail containing centrioles and flagella, which includes a 9 + 2 axonemal structure arrangement. The 9 + 2 structure arrangement consists of two central microtubules (CP) and nine peripheral microtubule doublets (OD) surrounded by outer dense fiber (ODF) and fibrous sheath (FS) (Silflow and Lefebvre, 2001; Avidor-Reiss and Fishman, 2019; Shen et al., 2019; Khan et al., 2021). In this study, we found some abnormalities in sperm morphology and some sperm flagellum lost, partially ODF, CP, and OD in *Cep72* KO mice (Figure 4D). These results suggest that compensatory mechanisms or redundant proteins may be present. Considering that there are approximately 70 family members of *Cep72* (Kumar et al., 2013), we explored the possible complementary mechanism by RNA-seq of *Cep72* KO testes. However, our data show no significant transcriptome changes (Supplementary Figure S2C). In particular, we did not observe differences in the transcripts of potential Cep72-binding proteins (Gheiratmand et al., 2019) and *Lrrc36*, which was the potential functionally redundant gene of *Cep72* (Stowe et al., 2012). Our data reveal only six transcripts that changed and differed significantly, of which *Gm29647* is a non-coding RNA and *Rps27a-ps2* and *Gm8430* are pseudo-genes. As coding genes, *Hbb-bt*, *Hba-a2*, and *Gm49527* have never been reported to be associated with spermatogenesis and fertility. Interestingly, mammalian oocytes lack centrosomes (Szollosi et al., 1972; Avidor-Reiss and Fishman, 2019). Previously, Cep72 has been identified as a member of the liquid-like meiotic spindle domain (LISD), a conserved subcellular structure in mammalian oocytes (So et al., 2019). However, our results show that the protein expression of Cep72 was barely detectable in mouse ovaries (Figure 1A), and *Cep72* KO female mice were fertile and had normal follicular development (Supplementary Figures S2A and B).

In summary, we identify Cep72 as a mouse testis-specific protein that does not affect mice fertility under physiological conditions. However, loss of Cep72 leads to partially abnormal spermiogenesis. Since environmental stresses could disturb fertility and spermatogenesis (Fon Tacer et al., 2019; Han et al., 2020), Cep72 may provide resistance to adverse environmental conditions in mouse testis. It has been reported that testis-specific genes such as *Sycp3* and *RAD21L1* are highly expressed in cancerous tissues (McFarlane and Makeman, 2017). This may be a reason that knockdown Cep72 could cause abnormalities in the cancer cell lines (Oshimori et al., 2009). Nevertheless, the Cep72 protein sequence homology of the mouse (NP_083235.3) shares only 67% of the sequence with human (NP_060610.2). Hence, Cep72 protein may have different expression patterns in humans and mice. Furthermore, the centrosome remodeling in spermatozoa in mice is different from most other mammals, including human (Avidor-Reiss et al., 2020). Therefore, whether the Cep72 regulates human spermatogenesis and fertility requires further in-depth studies.

## Data Availability

The datasets presented in this study can be found in online repositories. The names of the repository/repositories and accession number(s) can be found at: https://www.ncbi.nlm.nih.gov/, PRJNA835886.

## Supplementary material

Supplementary Figure S1 Testicular high expression of *Cep72* is mainly expressed in male germ cells of mice and humans. **(A)** RT-PCR results of *Cep72* expression levels in various tissues in mice. H_2_O was used as a negative control. *ACTB* was performed as an internal control. **(B)** GST-Cep72 (333-613aa)-8xHis antigen (66.5kD) was stained by Coomassie-blue (A lane, washing buffer A; B lane, washing buffer B; and E lane, elution buffer). **(C)** Histogram showing the expression profile of *Cep72* in mouse spermatogenic cells (GSE107644). **(D)** Histogram showing the expression profile of *Cep72* in human spermatogenic cells (GSE106487).

Supplementary Figure S2 Deficiency of *Cep72* does not affect fertility of female mice or global testicular transcript levels in male mice. **(A)** Fertility statistics of female *Cep72* KO mice. Data were provided as mean ± SEM, n = 4, ns: no significant difference. **(B)** H&E staining displaying the normal follicular development of 6-month-old female *Cep72* KO mice. **(C)** Scatter plot indicating the transcript levels of *Cep72* KO and WT mice. Differentially expressed genes (DEGs) with a log2 fold change (fold change ≥1, padj value ≤0.05) were shown in yellow plots. **(D)** Heatmap representing DEGs between *Cep72* KO and WT mice.
