# Supplementary material for: Loss of Cep72 affects the morphology of spermatozoa in mice
Source: Front Physiol. 2022 Oct 7;13:948965. doi: 10.3389/fphys.2022.948965 (PMC9585255; doi:10.3389/fphys.2022.948965)
Supplement: Supplementary file 1 [file Table1.DOCX]

The raw data were shared on Jianguoyun/Nutstore. The shareable link was

<https://www.jianguoyun.com/p/DVLGCuYQuubOChiGnMIEIAA>.

If the link did not be opened, please Sign in using the [zhenchen1988@whu.edu.cn](mailto:zhenchen1988@whu.edu.cn) (password is cz1988).
