# Supplementary figures and images for: Loss of Cep72 affects the morphology of spermatozoa in mice
Source: Front Physiol. 2022 Oct 7;13:948965. doi: 10.3389/fphys.2022.948965 (PMC9585255; doi:10.3389/fphys.2022.948965)

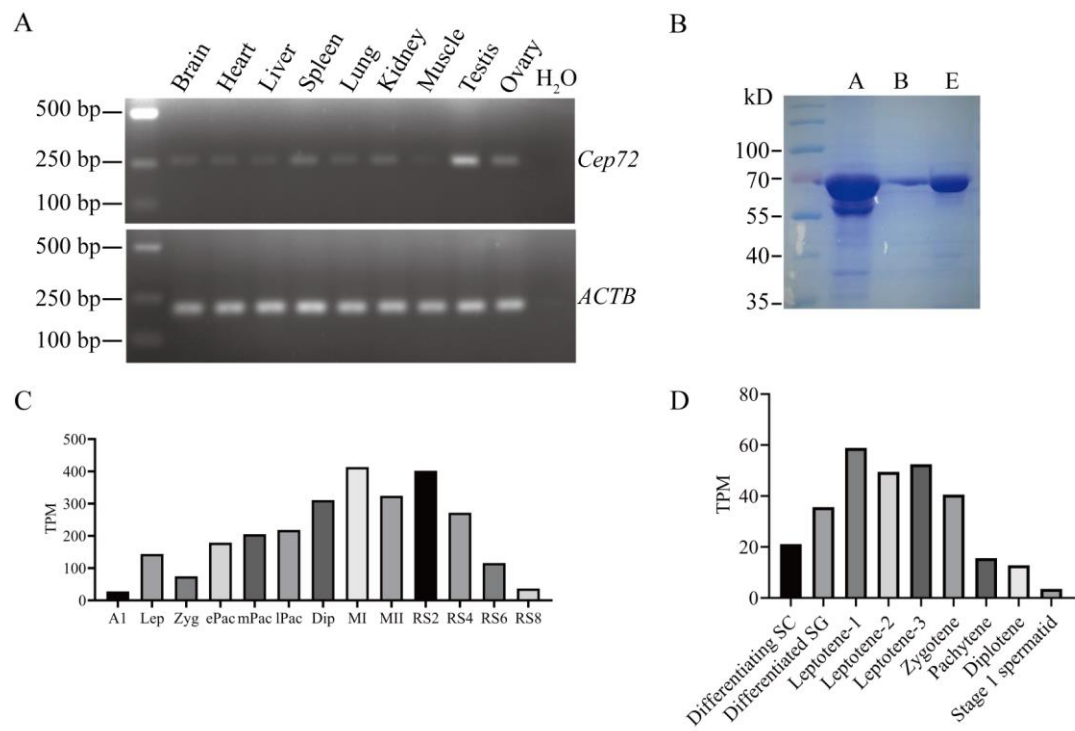

Figure S1

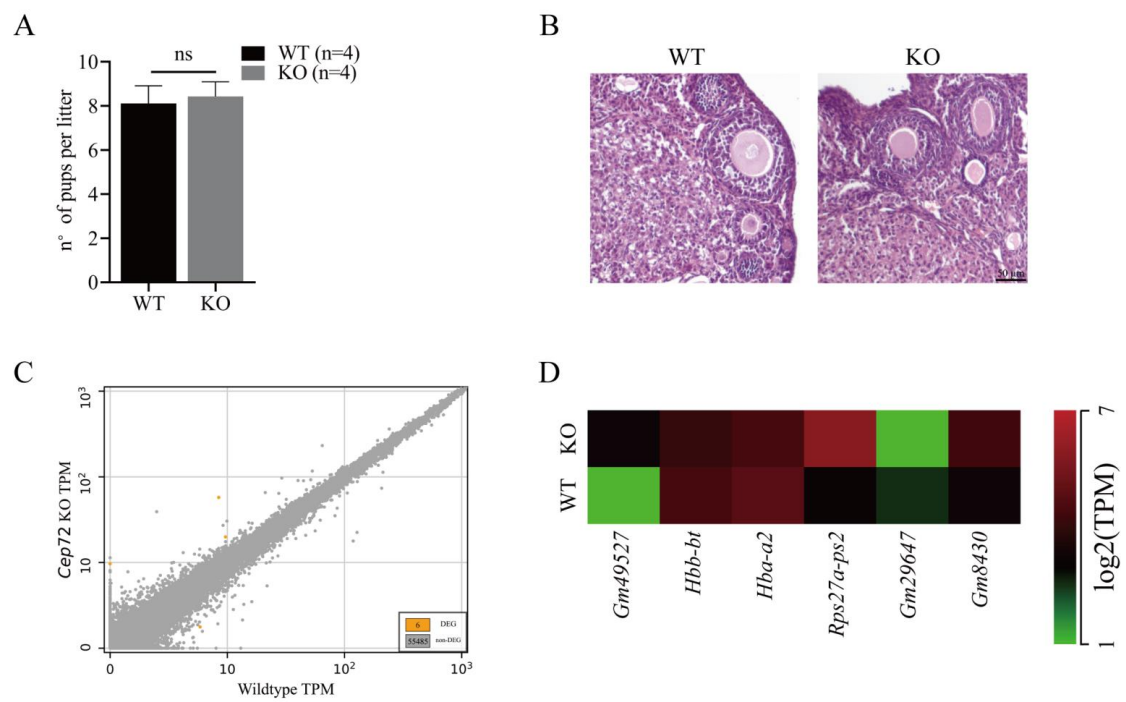

Figure S2

Supplement: Supplementary file 2 [file DataSheet1.PDF]
